# Reticulons 3 and 6 interact with viral movement proteins

**DOI:** 10.1111/mpp.13261

**Published:** 2022-08-20

**Authors:** Jens Tilsner, Verena Kriechbaumer

**Affiliations:** ^1^ Biomedical Sciences Research Complex School of Biology, Willie Russell Laboratories Fife UK; ^2^ Cell & Molecular Sciences The James Hutton Institute Dundee UK; ^3^ Endomembrane Structure and Function Research Group, Department of Biological and Medical Sciences Oxford Brookes University Oxford UK

**Keywords:** endoplasmic reticulum, FRET‐FLIM, plasmodesmata, protein–protein interaction, reticulon, viral movement protein

## Abstract

Plant reticulon (RTN) proteins are capable of constricting membranes and are vital for creating and maintaining tubules in the endoplasmic reticulum (ER), making them prime candidates for the formation of the desmotubule in plasmodesmata (PD). RTN3 and RTN6 have previously been detected in an *Arabidopsis* PD proteome and have been shown to be present in primary PD at cytokinesis. It has been suggested that RTN proteins form protein complexes with proteins in the PD plasma membrane and desmotubule to stabilize the desmotubule constriction and regulate PD aperture. Viral movement proteins (vMPs) enable the transport of viruses through PD and can be ER‐integral membrane proteins or interact with the ER. Some vMPs can themselves constrict ER membranes or localize to RTN‐containing tubules; RTN proteins and vMPs could be functionally linked or potentially interact. Here we show that different vMPs are capable of interacting with RTN3 and RTN6 in a membrane yeast two‐hybrid assay, coimmunoprecipitation, and Förster resonance energy transfer measured by donor excited‐state fluorescence lifetime imaging microscopy. Furthermore, coexpression of the vMP CMV‐3a and RTN3 results in either the vMP or the RTN changing subcellular localization and reduces the ability of CMV‐3a to open PD, further indicating interactions between the two proteins.

## Graphical abstract

Different vMPs are capable of interacting with RTN3 and RTN6. Coexpression of the vMP CMV‐3a and RTN3 results in proteins changing subcellular localization and reduces the ability of CMV‐3a to open plasmodesmata.

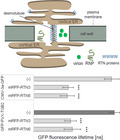

The plant endoplasmic reticulum (ER) is a multifaceted organelle with a variety of functions (Hawes et al., 2015). Apart from secretory protein production, folding, quality control (Brandizzi et al., 2003; Kriechbaumer & Brandizzi, 2020), and lipid biosynthesis (Wallis & Browse, 2010), it is also crucial for many other aspects of plant development, such as oil and protein body formation (Herman, 2008; Huang, 1996; Schmidt & Herman, 2008) and auxin regulation (Friml & Jones, 2010; Kriechbaumer et al., 2016). The plant cortical ER network is also involved in protein trafficking (Vitale & Denecke, 1999) and pathogen responses (for review see Beck et al., 2012; Pattison & Amtmann, 2009).

The ER network forms a polygonal structure of tubules and cisternae (Sparkes, Runions, et al., 2009a; Sparkes, Frigerio, et al., 2009b). The reticulon (RTN) proteins are crucial for the tubulation of the ER and are essential in maintaining the tubular ER network (Sparkes et al., 2010; Tolley et al., 2008, 2010). RTNs are integral membrane proteins containing an RTN homology domain composed of four transmembrane domains forming a “W” shape in the membrane with the C‐ and N‐termini of the protein facing the cytosol (Figure 1). RTNs are capable of dimerization and oligomerization, which leads to ER membrane tensions and thereby induces membrane curvature (Sparkes et al., 2010).

**FIGURE 1:**
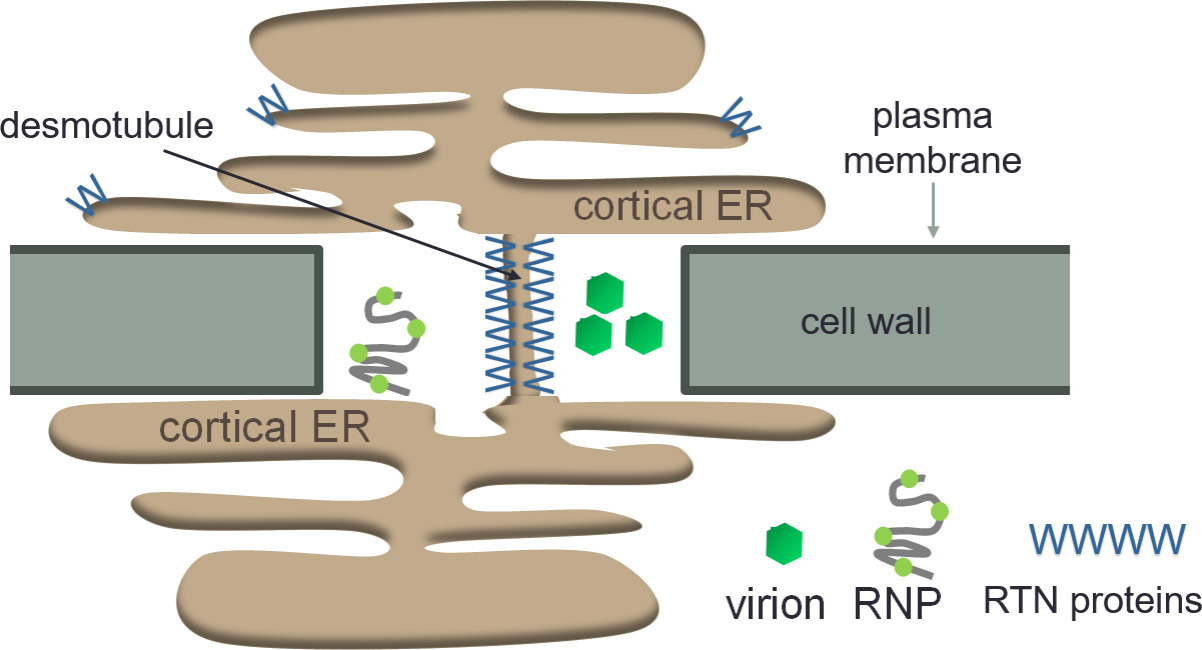
Schematic representation of the endoplasmic reticulum (ER) crossing the cell wall through plasmodesmata. The cortical ER connects two cells via the desmotubule through plasmodesmata. RTNs tubulate the ER membrane and potentially the desmotubule. Virions or viral ribonucleoprotein (RNP) complexes move from cell to cell through plasmodesmata.

The ability of RTNs to constrict membranes is of great interest for cell plate development and the formation of plasmodesmata (PD) (Knox et al., 2015; Kriechbaumer et al., 2015). The formation of PD requires extensive remodelling of the ER into a hyperconstricted tubule termed the desmotubule that runs through the PD pore (Blackman et al., 1999) (Figure 1). The desmotubule is only approximately 15–20 nm in diameter and as such is one of the most constricted membrane structures to be found in nature (Tilsner et al., 2011). As RTNs are able to hyperconstrict tubules (Sparkes et al., 2010), they are prime candidates for creating the tightly furled desmotubule. Two of the RTNs present in a PD proteome generated from *Arabidopsis* suspension culture cells, RTN3 and RTN6 (Fernandez‐Calvino et al., 2011), are present in primary PD at cytokinesis (Knox et al., 2015), and in a coimmunoprecipitation screen they were shown to interact with a variety of ER, PD, and plasma membrane (PM) proteins (Kriechbaumer et al., 2015). Hence it was suggested that RTNs form protein complexes with proteins in the PD PM and desmotubule to stabilize desmotubule constriction and regulate PD aperture (Knox et al., 2015; Kriechbaumer et al., 2015; Tilsner et al., 2011).

Many of the proteins interacting with RTNs have also been shown to be targeted by viral movement proteins (vMPs). Examples include the remorin proteins REM1.2 and REM1.3 (Borner et al., 2005), synaptotagmins (SYTs) 1/A and 7 (Ishikawa et al., 2020; Lewis & Lazarowitz, 2010; Uchiyama et al., 2014), the atlastin homologue ROOT HAIR DEFICIENT 3 (RHD3; Feng et al., 2016), and Vesicle‐Associated Protein 27 (VAP27; Carette et al., 2002).

vMPs enable the transport of viruses through PD (Tilsner et al., 2014) and often are either ER‐integral membrane proteins or interact with the ER (Krishnamurthy et al., 2003; Peiró et al., 2014; Vilar et al., 2002). vMPs of potato virus X have been shown to be enriched in desmotubules in *Nicotiana benthamiana* (Tilsner et al., 2013). Some vMPs can themselves hyperconstrict ER membranes or preferentially localize to RTN‐containing tubules (Lazareva et al., 2021; Lee et al., 2010).

Here we show that RTN3 and RTN6 are capable of direct interactions with various vMPs, including both ER‐integral and peripherally membrane‐associated vMPs. The methods are described in Text S1.

Protein–protein interactions between RTN3 and RTN6 and a selection of vMPs were first tested using a yeast mating‐based split‐ubiquitin system (mbSUS; Asseck et al., 2018). We initially focused on vMPs that are known to be ER‐integral: potato virus X triple gene block 2 (PVX‐TGB2), potato virus X triple gene block 3 (PVX‐TGB3), barley stripe mosaic virus triple gene block 2 (BSMV‐TGB2), and potato mop‐top virus triple gene block 2 (PMTV‐TGB2) (Haupt et al., 2005; Krishnamurthy et al., 2003; Mitra et al., 2003; Torrance et al., 2006) (Figure 2). Unlike conventional yeast two‐hybrid systems, mbSUS allows for interaction studies of full‐length membrane proteins in a native cellular setting. mbSUS uses the ubiquitin proteasome pathway to release an artificial transcription factor that results in the activation of reporter genes to visualize protein–protein interactions. N‐ and C‐terminal ubiquitin moieties (Nub and Cub, respectively) are brought into close proximity when they are fused to interacting proteins, resulting in reconstitution of a functional ubiquitin molecule. For mbSUS, Nub is mutated to reduce its affinity for Cub, thereby preventing spontaneous reassembly with Cub (Asseck et al., 2018).

**FIGURE 2:**
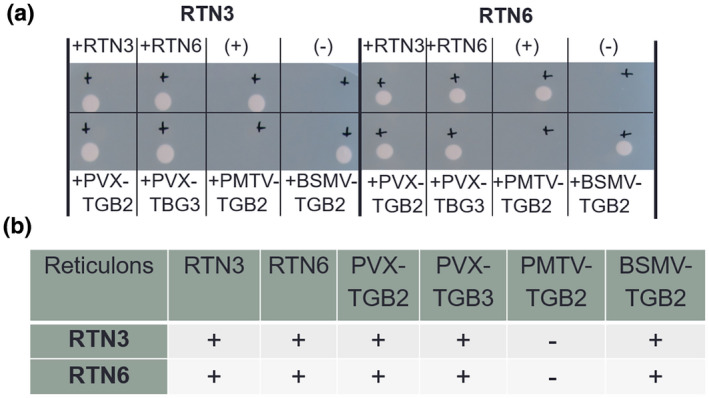
Interactions between RTN3 and RTN6 with viral movement proteins (vMPs) using a mating‐based split‐ubiquitin system. (a) Yeast colony growth indicates a protein–protein interaction for the protein pair; no growth indicates no interaction. Interactions were confirmed for the controls (top row): RTN3/RTN3, RTN3/RTN6, RTN6/RTN3 and RTN6/RTN6. Positive and negative controls for the yeast system are also shown (top row “(+)” and “(−)”). For the interactions of RTN3 and RTN6 with vMPs, positive interactions were shown for both RTN3 and RTN6 with potato virus X triple gene block 2 (PVX‐TGB2), potato virus X triple gene block 3 (PVX‐TGB3), and barley stripe mosaic virus triple gene block 2 (BSMV‐TGB2), but not for RTN3 and RTN6 with potato mop‐top virus triple gene block 2 (PMTV‐TGB2). (b) Summary of the interactions, with “+” indicating an interaction and “−“ indicating no interaction.

Standard controls for the mbSUS system were included. NubWT was used as a positive control NubWT (+), as the nonmutated fragment can bind to Cub without other interacting proteins present; an empty vector (−) was used as a negative control (Figure 2a). In addition, interactions between the two RTNs as homo‐ or heterodimers were used as positive controls (Kriechbaumer et al., 2015; Sparkes et al., 2010). Colony growth (Figure 2a) indicates an interaction between the proteins tested. The absence of a colony suggests that no protein interaction has taken place (summarized in Figure 2b). Colony growth indicated protein–protein interactions for both RTN3 and RTN6 with PVX‐TGB2, PVX‐TGB3 and BSMV‐TGB2. No interactions were shown in this system for RTN3 and RTN6 with PMTV‐TGB2.

To further test such interactions between RTNs and vMPs in vivo in a plant background, Förster resonance energy transfer (FRET) measured by donor excited‐state fluorescence lifetime imaging microscopy (FLIM) (Becker, 2012; Kriechbaumer et al., 2015; Kriechbaumer & Botchway, 2018; Schoberer & Botchway, 2014) was used. FRET‐FLIM measures the reduction in the lifetime of green fluorescence protein (GFP; donor) fluorescence when an acceptor fluorophore (mRFP) is within a distance of 1–10 nm and therefore enables FRET to occur, which indicates a physical interaction between the two proteins (Kriechbaumer et al., 2015; Sparkes et al., 2010). In the FRET‐FLIM assay, RTN3 or RTN6 was transiently expressed as an mRFP fusion protein (acceptor) in tobacco leaf epidermal cells together with the vMP as a GFP fusion protein (donor). In addition to PVX‐TGB2 and PMTV‐TGB2 previously tested by mbSUS, we chose two vMPs for which ER association is less clear, tobacco mosaic virus 30k (TMV‐30k), a peripheral membrane protein that causes ER disruption (Peiró et al., 2014; Reichel & Beachy, 1998), and cauliflower mosaic virus 3a (CMV‐3a), whose membrane association has not been characterized (Canto & Palukaitis, 2005). FRET‐FLIM interactions are shown in Table 1 and Figures 3 and S1. CMV‐3a‐GFP, GFP‐PVX‐TGB2, GFP‐PMTV‐TGB2 or TMV‐30k‐GFP expression without acceptor were used as negative controls. The vMP‐GFP fusions alone showed fluorescence lifetimes in the range of 2.37 to 2.41 ns. Excited‐state lifetimes determined for all four vMPs coexpressed with RTN3 or RTN6 varied from 2.04 to 2.19 ns (Table 1, Figure 3), which was statistically significantly different from that of the vMP GFP fusion alone in all cases. Coexpression of mRFP‐RTN3 or mRFP‐RTN6 resulted in a GFP lifetime reduction of 0.22–0.33 ns (Table 1); a reduction in excited‐state lifetime of 0.2 ns or more is indicative of energy transfer (Stubbs et al., 2005). As a control for the setup and system, FRET‐FLIM was also carried out with GFP‐RTN3 as a donor. Interactions here were shown with both mRFP‐RTN3 and mRFP‐RTN6, with GFP lifetime reductions comparable to those observed for vMPs (Table 1), as described in a previous work (Kriechbaumer et al., 2015). These interactions are not artificially enforced by protein overexpression and membrane crowding as shown by the lack of interaction between RTN3 and RTN6 with Annexin 4 (ANNAT4, Figure S2).

**TABLE 1:** Fluorescence lifetimes in FRET‐FLIM analysis

Donor (GFP)	Acceptor (mRFP)	Average GFP fluorescence lifetime [ns ± *SD*]	Δ
CMV‐3a	(−)	2.37 ± 0.05	
CMV‐3a	+RTN3	2.05 ± 0.04	0.32
CMV‐3a	+RTN6	2.04 ± 0.05	0.33
PVX‐TGB2	(−)	2.39 ± 0.04	
PVX‐TGB2	+RTN3	2.07 ± 0.04	0.32
PVX‐TGB2	+RTN6	2.08 ± 0.06	0.31
PMTV‐TGB2	(−)	2.41 ± 0.07	
PMTV‐TGB2	+RTN3	2.12 ± 0.05	0.29
PMTV‐TGB2	+RTN6	2.19 ± 0.04	0.22
TMV‐30k	(−)	2.40 ± 0.03	
TMV‐30k	+RTN3	2.09 ± 0.05	0.31
TMV‐30k	+RTN6	2.08 ± 0.04	0.32
RTN3	(−)	2.42 ± 0.02	
RTN3	+RTN3	2.18 ± 0.02	0.24
RTN3	+RTN6	2.20 ± 0.02	0.22

*Notes*: Interactions between the viral movement proteins cauliflower mosaic virus 3a (CMV‐3a), potato virus X triple gene block 2 (PVX‐TGB2), potato mop‐top virus triple gene block 2 (PMTV‐TGB2) and tobacco mosaic virus 30k (TMV‐30k) with the reticulon proteins RTN3 and RTN6 were analysed. Donor and acceptor protein constructs are listed together with the average fluorescence lifetime (in ns) for the donor fluorophore and the *SD* for each combination. The difference between control and test samples was calculated (Δ). It was previously reported that a reduction in excited‐state lifetime of 0.2 ns is indicative of energy transfer (Stubbs et al., 2005). For each combination, at least three biological samples with a minimum of 10 technical replicates were used for analysis. Negative (GFP‐RTN3 alone) and positive controls (RTN3 with RTN3 and RTN3 with RTN6) are included.

**FIGURE 3:**
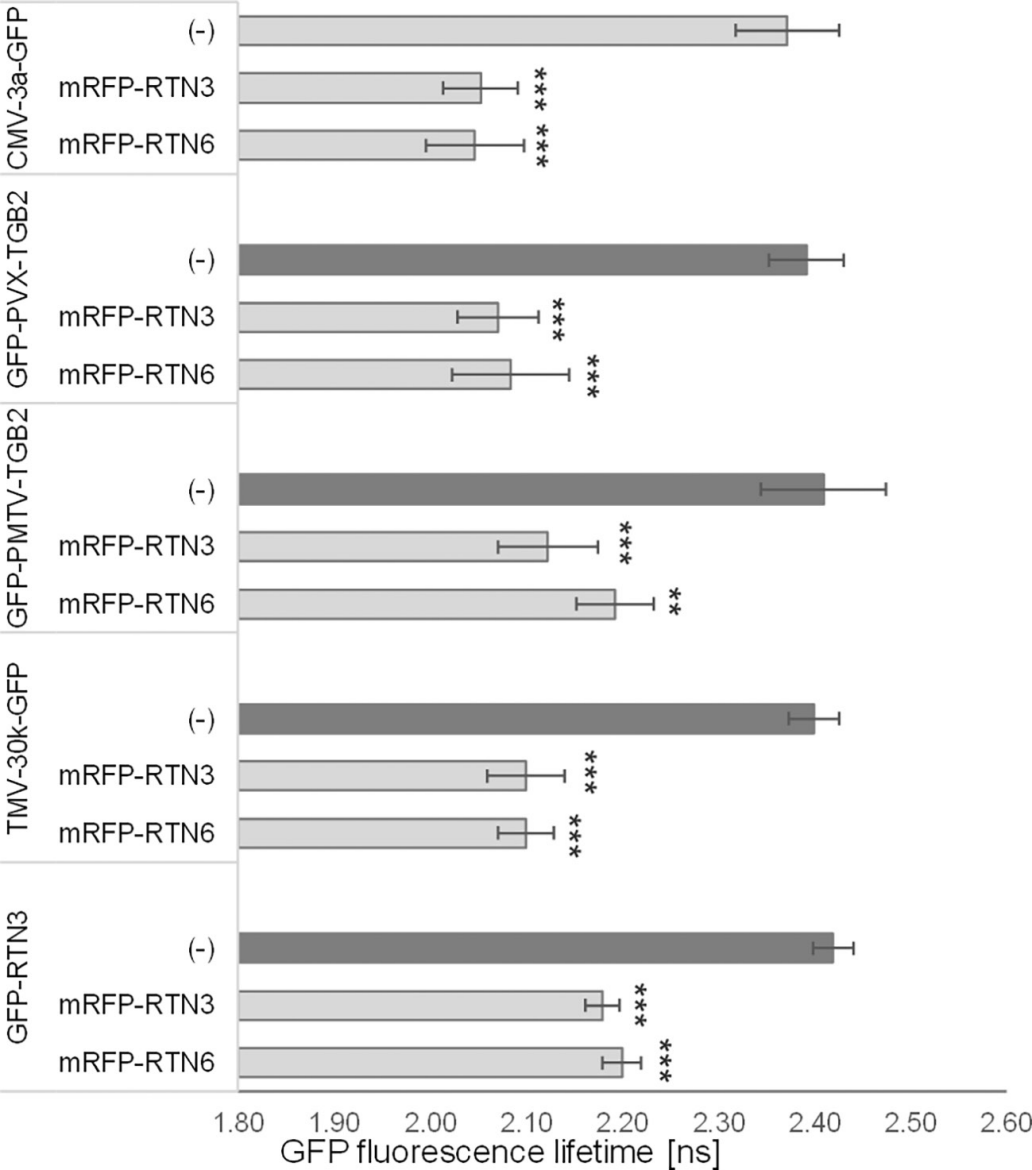
Fluorescence lifetimes in FRET‐FLIM interactions. Interactions between the viral movement proteins cauliflower mosaic virus 3a (CMV‐3a), potato virus X triple gene block 2 (PVX‐TGB2), potato mop‐top virus triple gene block 2 (PMTV‐TGB2) and tobacco mosaic virus 30k (TMV‐30k) with RTN3 and RTN6 were analysed. RTN3–RTN3 homo‐ and RTN3–RTN6 heterotypic interactions were included as positive controls. The bar graphs represent average fluorescence lifetimes (ns) and the corresponding *SD* values for the green fluorescent protein (GFP) donors CMV‐3a‐GFP, GFP‐PVX‐TGB2, GFP‐PMTV‐TGB2, TMV‐30k‐GFP, and GFP‐RTN3. The data show the lifetimes of CMV‐3a‐GFP, GFP‐PVX‐TGB2, GFP‐PMTV‐TGB2, TMV‐30k‐GFP, and GFP‐RTN3 without interaction partners (dark grey bars) compared to the lifetimes of these donors coexpressed with mRFP‐RTN3 or mRFP‐RTN6 (light grey bars). Excited‐state lifetimes of 0.2 ns shorter than those of the GFP donor alone indicate protein–protein interactions (Stubbs et al., 2005). This is the case for all interactions shown here, indicating that CMV‐3a‐GFP, GFP‐PVX‐TGB2, GFP‐PMTV‐TGB2, TMV‐30k‐GFP and GFP‐RTN3 interact with both mRFP‐RTN3 and mRFP‐RTN6. Significance was analysed by the Kruskal–Wallis test (*p < 0.05, *p < 0.01, ***p < 0.001). *n* = 3 with at least 10 technical replicates each.

In contrast to the mbSUS system, the in planta FRET‐FLIM analysis showed interactions of GFP‐PMTV‐TGB2 with mRFP‐RTN3 and mRFP‐RTN6. To clarify this discrepancy, GFP‐PMTV‐TGB2 was coexpressed with mRFP‐RTN3 or mRFP‐RTN6, and coimmunoprecipitation followed by western blotting was carried out (Figure S3). Here, both RTN3 and RTN6 precipitated with PMTV‐TGB2, indicating that these proteins do indeed interact. It is unclear why this interaction did not take place in the yeast system but mistargeting of proteins, lower expression levels, or differences of membrane composition and environment in the heterologous system are possible explanations.

To investigate the interactions between the (*Arabidopsis*) RTNs and vMPs in *Arabidopsis thaliana*, rather than a heterologous system, stable transgenic plants were generated expressing mRFP‐RTN3 together with CMV‐3a‐GFP, because cucumber mosaic virus can infect *Arabidopsis*. Expression of both proteins was observed in the hypocotyl and the root in approximately 60% of all cells in T_1_ plants 5 days after germination but could only be observed in fewer than 1% of the cells 10 days after germination and was not detectable in the T_2_ generation. Interestingly though, in all cells displaying both constructs the localization of either one or the other of the two proteins changed (Figure 4). CMV‐3a‐GFP alone labelled PD at the cell periphery (Figures 4a and S4; Canto et al., 1997) and mRFP‐RTN3 alone labelled the whole ER (Figures 4c and S4). In coexpressing cells, either CMV‐3a‐GFP was present in both the ER and PD, interestingly remaining in a punctate structure in the ER (Figures 4b and S4), or, in rarer cases (c.5%), RFP‐RTN3 was detectable in PD but not anymore throughout the ER (Figure 4d). This change in localization indicates that the two proteins interact potentially rather strongly and therefore are targeted together to the same localization. The coexpression of CMV‐3a‐GFP with mRFP‐RTN3 was also tested by transient expression in tobacco leaf cells (Figure S5). Here CMV‐3a‐GFP remained restricted to PD in 68% of the cells visualized (Figure S5a A–C; *n* = 4 with 10 cells each). However, in 32% of cells, CMV‐3a‐GFP additionally localized to puncta on the ER (Figure S5b D–F), similar to stable expression in *Arabidopsis*.

**FIGURE 4:**
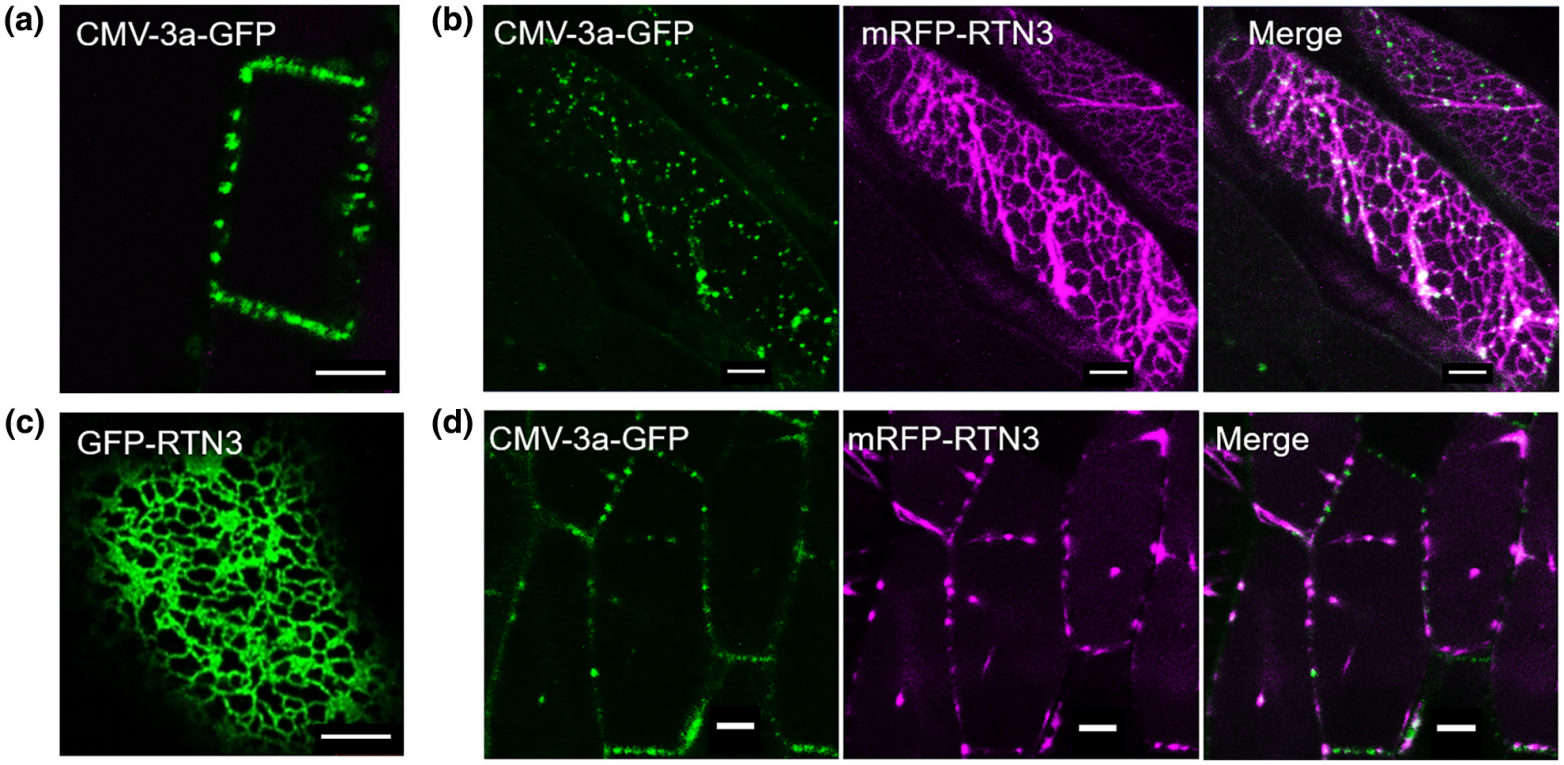
Localization of CMV‐3a‐GFP and mRFP‐RTN3 in stably transformed *Arabidopsis* cells. Coexpression of the viral movement protein CMV‐3a‐GFP and the reticulon protein mRFP‐RTN3 results in a change of subcellular localization for one of the proteins. Mainly, CMV‐3a‐GFP is no longer restricted to plasmodesmata (a) but also labels the endoplasmic reticulum in puncta (b). In rarer cases, mRFP‐RTN3 no longer labels the whole endoplasmic reticulum (c) but is restricted to plasmodesmata (d). Scale bars = 5 μm. See Figure S4 for a more detailed presentation of protein localizations at the cell surface and median.

To evaluate a potential effect of this interaction on vMP function, the ability of CMV‐3a to increase the PD size exclusion limit was analysed with and without coexpression of RTN3 (Figure 5). Based on the methodology in Perraki et al. (2014), diffusion of cytosolic GFP into neighbouring cells was analysed when coexpressed with CMV‐3a, RTN3 or both (Figure 5). Infiltration of cytosolic GFP *Agrobacterium* suspension at a low optical density resulted in single cells expressing GFP (Figure 5). Cell clusters where the GFP diffused into three or more cells were counted and normalized to GFP alone controls (100%). When coexpressed with CMV‐3a, GFP diffusion into adjacent cells increased to 144 ± 11%. However, when coexpressed with both CMV‐3a and RTN3, diffusion decreased relative to the effect of CMV‐3a alone (114 ± 13%), indicating that the ability of CMV‐3a to open PD was reduced. Coexpression of cytosolic GFP with RTN3 had no significant effect on diffusion (91 ± 13%). It was previously suggested that vMPs interact with RTN proteins and dislodge them from the desmotubule, thereby enabling relaxation of the desmotubule and viral movement (Tilsner et al., 2011). The overexpression of RTN3 might therefore reinstate desmotubule constriction and/or remove a sufficient amount of CMV3a from the PD.

**FIGURE 5:**
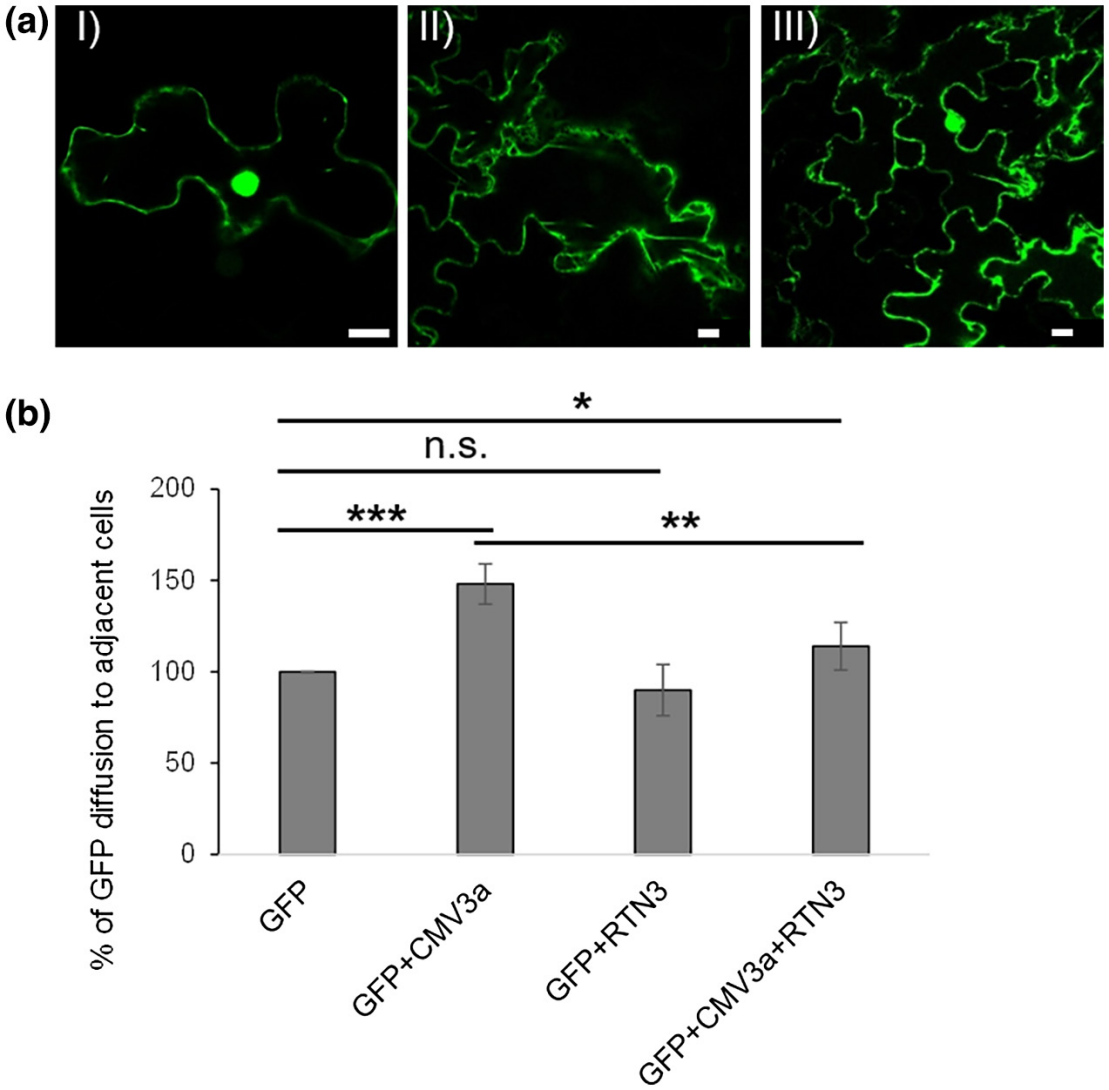
The ability of CMV‐3a to open plasmodesmata is reduced by RTN3. Single cells expressing cytosolic green fluorescent protein (GFP) were observed 2 days after infiltration with a diluted *Agrobacterium* culture (optical density at 600 nm = 0.01) into tobacco leaves (I). Diffusion of cytosolic GFP to three (II) or more (III) adjacent cells was measured with GFP alone, GFP with CMV‐3a‐mRFP, GFP with RFP‐RTN3, and GFP with CMV‐3a‐mRFP and RTN3. Example images are shown (a). Clusters with GFP labelling three or more adjacent cells were counted and normalized to GFP alone (100%). Bars represent averages and error bars represent standard deviations (b). Significance was analysed by the Kruskal–Wallis test (*p < 0.05, *p < 0.01, ***p < 0.001; n.s. = not significant). *n* = 4 with at least 10 technical replicates each. Scale bars = 10 μm.

Taken together, various vMPs can interact with plant RTN3 and RTN6 as shown in a membrane yeast two‐hybrid approach (Figure 2), by FRET‐FLIM (Figure 3), by coimmunoprecipitation (Figure S3), and in the change of localization in stable coexpression in *Arabidopsis* plants (Figure 4), as well as in transient expression in tobacco plants (Figure S5).

RTN3 and RTN6 have previously been shown to interact with a variety of proteins in the PD proteome, PM proteins, and ER–PM contact site components (Kriechbaumer et al., 2015). RTN3 and RTN6 have also been localized to PD (Knox et al., 2015) and RTN proteins have been suggested to be involved in regulating PD aperture directly by maintaining constriction of the desmotubule (Knox et al., 2015; Tilsner et al., 2011).

In mammalian cells, RTN proteins have been shown to be involved in the formation of ER–PM and ER–mitochondrial membrane contact sites (Caldieri et al., 2017), and in *Arabidopsis*, RTNs interact with ER–PM contact site proteins such as SYT1/A and 7 and VAP27 (Ishikawa et al., 2020; Kriechbaumer et al., 2015; Levy et al., 2015; Pérez‐Sancho et al., 2015; Wang et al., 2014). RTNs have also been suggested to play a role in the assembly of the brome mosaic virus replication complex by stabilizing positive membrane curvature at the openings of ER‐derived spherules holding the replication complexes (Diaz & Ahlquist, 2012; Diaz et al., 2010). Additionally, formation of viral replication complexes requires delivery of specific lipids, likely at least in part through nonvesicular intermembrane exchange at membrane contact sites (Barajas et al., 2014; Nagy et al., 2016).

Hence, it is suggested that RTNs can be involved in PD regulation not only by actively constricting the desmotubule but also by bringing other proteins to membrane contact sites (Levy & Tilsner, 2020; Tilsner et al., 2011).

Although most vMPs are dispensable for virus replication, they can participate in the formation of viral replication complexes, the membrane‐derived structures supporting virus replication (Más & Beachy, 1999; Tilsner et al., 2012), and recruit replication complexes to PD potentially to increase the specificity and speed of virus transport (Levy et al., 2015; Levy & Tilsner, 2020; Tilsner et al., 2013). Thus, vMP interactions with RTNs may serve multiple purposes during infection, including modification of the architecture and composition of cellular membranes to facilitate virus replication, targeting to PD via the desmotubule, and direct or indirect modification of PD. Future research will identify what specific roles RTN3 and RTN6 play in the infection of the different viruses whose vMPs were included in this study.

## Supporting information


**Figure S1** FRET‐FLIM data for interactions. Viral movement proteins CMV‐3a, PVX‐TGB2, PMTV‐TGB2, and TMV‐30k were expressed in tobacco leaf cells as donors. FRET‐FLIM interactions with mRFP‐RTN3 and mRFP‐RTN6 or without an acceptor (−) as a negative control are shown. (a) The raw FRET‐FLIM data. (b) Pseudo‐coloured lifetime maps display the lifetime values for each point within the region of interest. (c) The distribution of lifetimes across the entire image with blue shades representing longer GFP fluorescence lifetimes than green ones. (d) Representative decay curves of a single point (indicated by blue cross‐hairs in a,b) with an optimal single exponential fit, where χ^2^ values from 0.9 to 1.2 are considered an excellent fit to the data points

**Figure S2** FRET‐FLIM control with noninteracting endoplasmic reticulum (ER) proteins. Interactions were tested between RTN6 and RTN3, respectively, with the ER‐localized protein Annexin 4 (ANNAT4). (a) Example data for GFP‐RTN3 alone as a negative control or GFP‐RTN3 with mRFP‐ANNAT4. Average lifetimes ± *SD* are given in brackets. (b) Statistical analysis for GFP‐RTN3 and GFP‐RTN6 without and with mRFP‐ANNAT4. ANNAT4 shows no interaction with RTN3 or RTN6 (n.s.). Significance was analysed by the Kruskal–Wallis test (*p < 0.05, *p < 0.01, ***p < 0.001)

**Figure S3** Coimmunoprecipitation assay to determine protein–protein interactions for PMTV‐TGB2 with RTN3 and RTN6. mRFP‐RTN3 or mRFP‐RTN6 was expressed in tobacco epidermal leaf cells alone or together with GFP‐PMTV‐TGB2. Coimmunoprecipitation was carried out using an anti‐GFP antibody linked to agarose (GFP‐Trap; Chromotek). As a negative control mRFP‐RTN3 and mRFP‐RTN6 were incubated with GFP‐Trap beads without GFP‐PMTV‐TGB2. Proteins were separated by 12% SDS‐PAGE and probed with anti‐RFP antibodies (a) to detect the prey (RTN3/RTN6) as well as anti‐GFP antibodies (b) to show that the bait had been precipitated (PMTV‐TBG2). (c) The input for the prey mRFP‐RTN3/6

**Figure S4** Localization of CMV‐3a and RTN3 individually and together in *Arabidopsis* cells. Stable *Arabidopsis* plants expressing CMV‐3a‐GFP or GFP‐RTN3 were created and the localization of both proteins throughout the cell was visualized. Representative example stacks together with 3D reconstructions are shown. CMV‐3a‐GFP alone localized to plasmodesmata (PD) and GFP‐RTN3 alone to the endoplasmic reticulum (ER). Slice depth is given. For comparison, a representative cell expressing both CMV‐3a and RTN3 is shown. Here CMV‐3a‐GFP was found in PD but also distributed in puncta over the ER. Size bars = 5 μm

**Figure S5** Transient coexpression of CMV‐3a‐GFP and mRFP‐RTN3 in tobacco epidermal leaf cells. CMV‐3a‐GFP (green) was coexpressed in tobacco epidermal leaf cells with mRFP‐RTN3 (magenta) and the localization was analysed in *n* = 4 biological replicas with 10 cells each. Representative example images are shown in (a–f). In this system, CMV‐3a‐GFP localized to plasmodesmata (PD) (a,b) only but not the endoplasmic reticulum (ER) (c) in 68% of cells visualized. In 32% of cells, CMV‐3a‐GFP was additionally also localized in dots on the ER (d,e), labelling both the peripheral ER and some PD (f). Size bars = 5 μm for (a–d) and = 10 μm for (f)

**Text S1** Materials and methods used in this work

## Data Availability

The data that support the findings of this study are available from the corresponding author upon reasonable request.
