# Supplementary material for: Reticulons 3 and 6 interact with viral movement proteins
Source: Mol Plant Pathol. 2022 Aug 20;23(12):1807–14. doi: 10.1111/mpp.13261 (PMC9644274; doi:10.1111/mpp.13261)

Supplementary Figure S1a

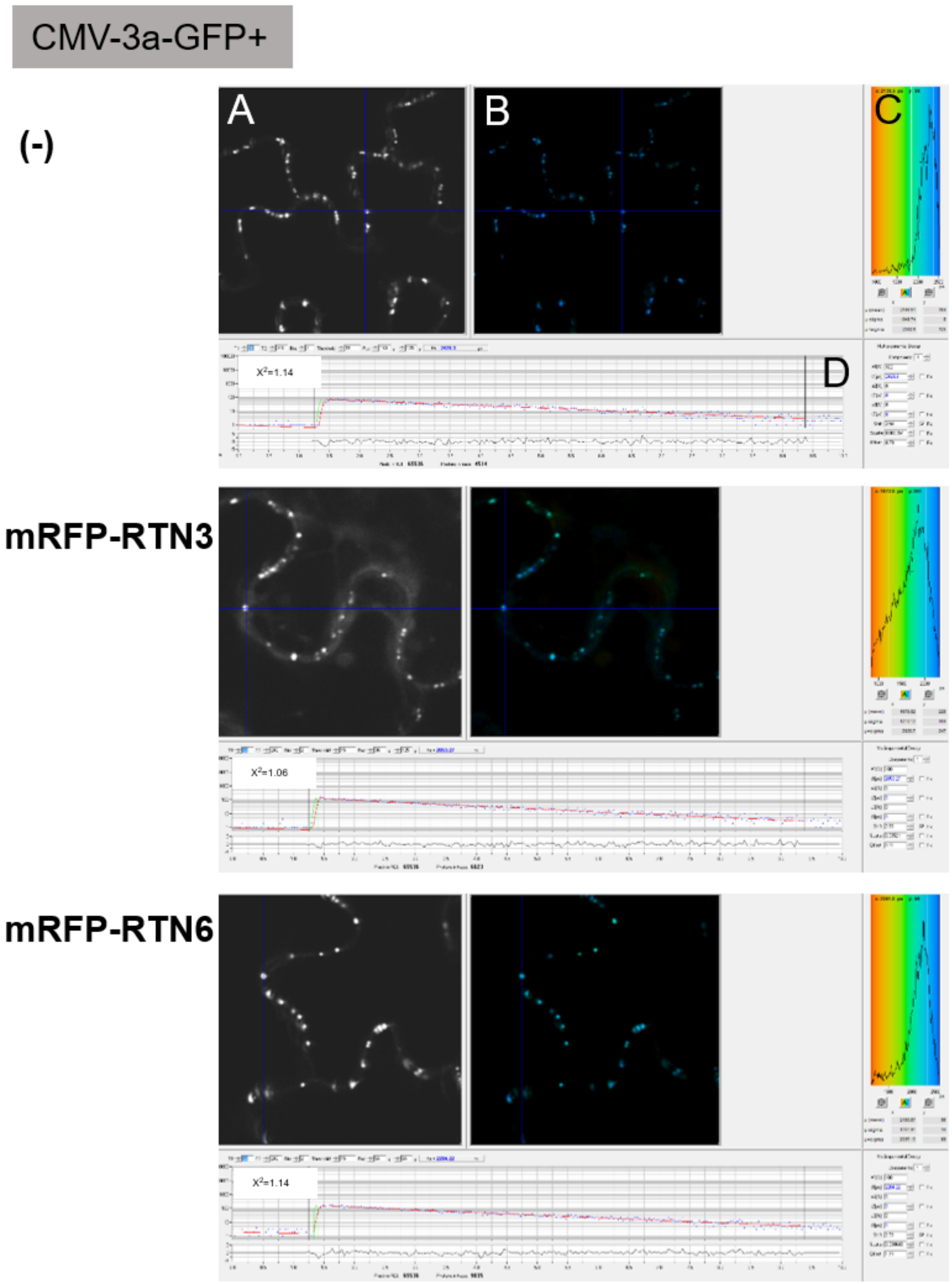

Supplementary Figure S1: FRET-FLIM data for interactions.

vMPs CMV-3a, PVX-TGB2, PMTV-TGB2, TMV-30k, respectively, were expressed in tobacco leaf cells as a donor. FRET-FLIM interactions without an acceptor (-) as a negative control, and with mRFP-RTN3 and mRFP-RTN6, respectively, are shown. (A) depicts the raw FRET-FLIM data. In (B), pseudo-coloured lifetime maps display the lifetime values for each point within the region of interest. The distribution of lifetimes across the entire image is shown in (C) with blue shades representing longer GFP fluorescence lifetimes than green ones. (D) shows representative decay curves of a single point (indicated by blue cross-hairs in A, B) with an optimal single exponential fit, where $\chi 2$ values from 0.9 to 1.2 are considered an excellent fit to the data points.

Supplementary Figure S1b

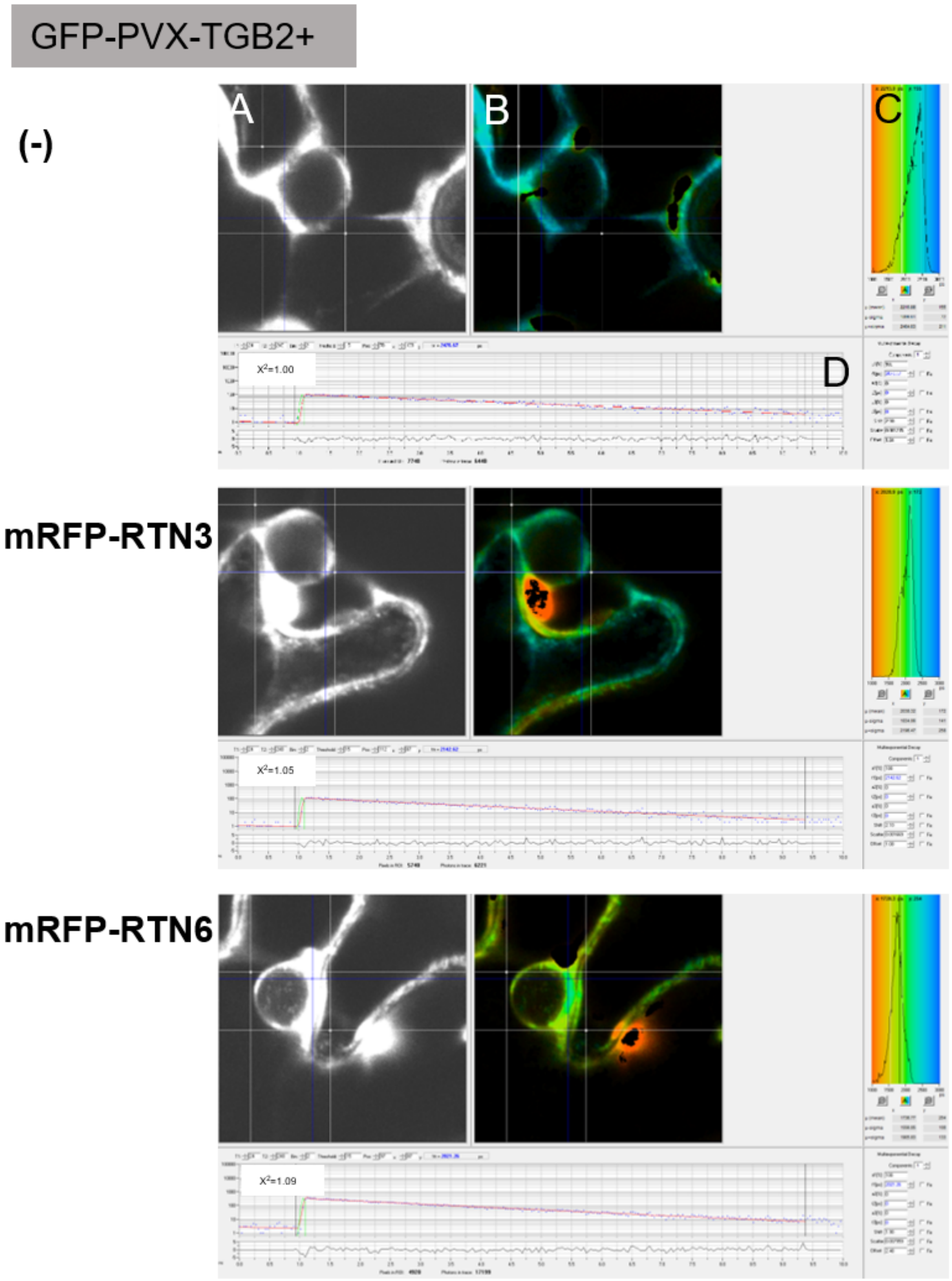

Supplementary Figure S1c

GFP-PMTV-TGB2+

(-)

mRFP-RTN3

mRFP-RTN6

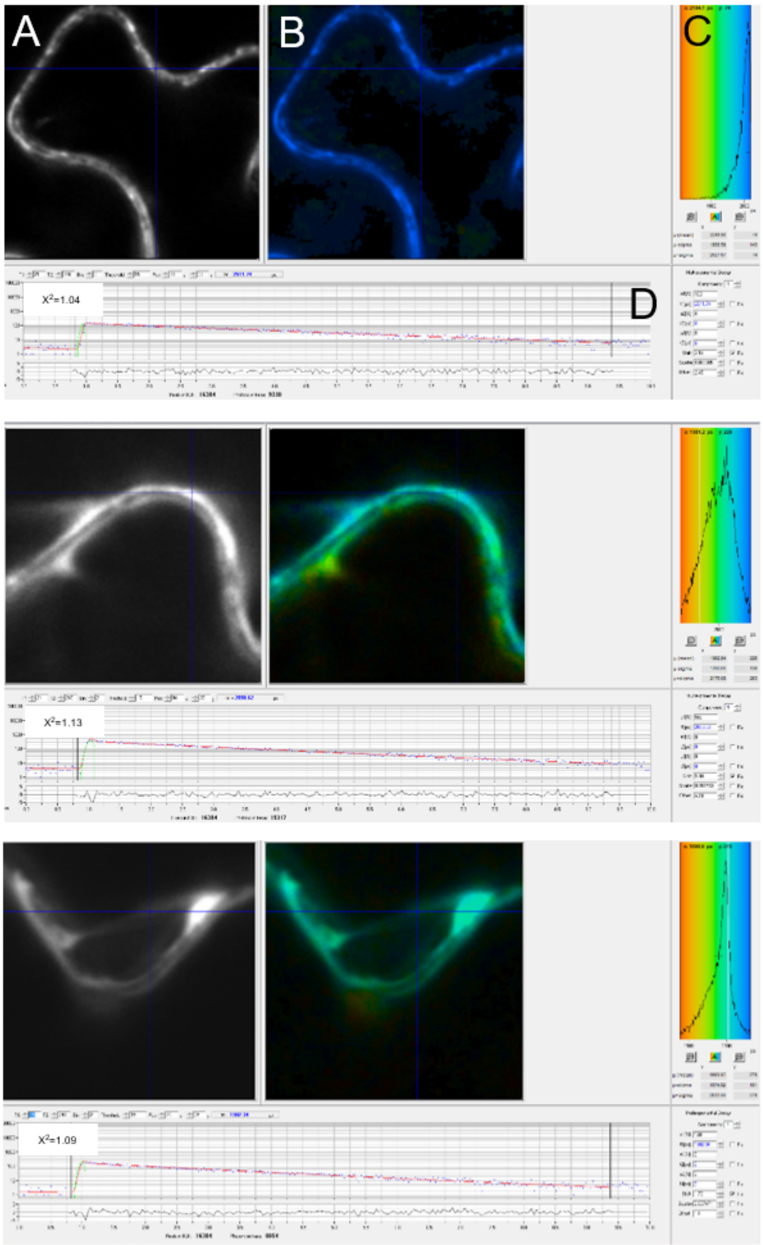

Supplementary Figure S1d

TMV-30k-GFP+

(-)

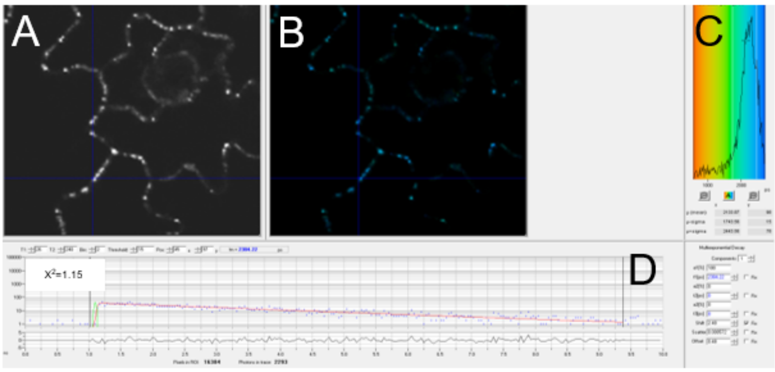

mRFP-RTN3

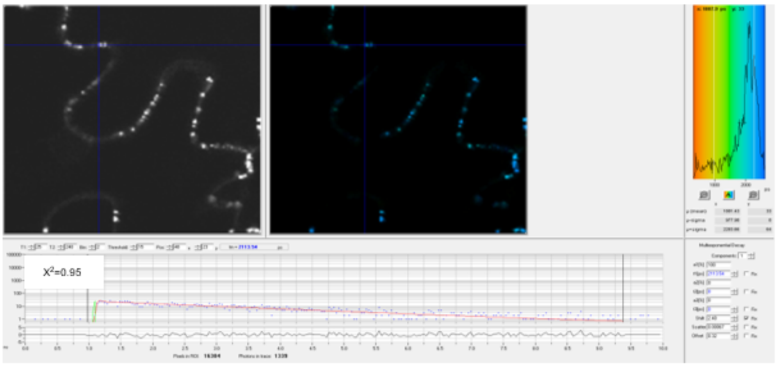

mRFP-RTN6

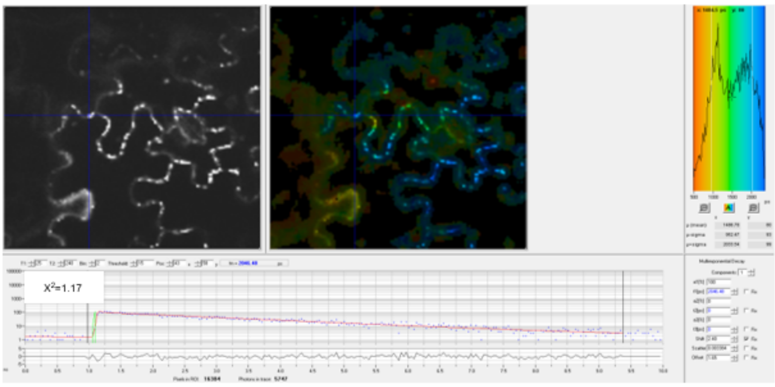

Supplement: Supplementary file 1 — Figure S1 FRET‐FLIM data for interactions. Viral movement proteins CMV‐3a, PVX‐TGB2, PMTV‐TGB2, and TMV‐30k were expressed in tobacco leaf cells as donors. FRET‐FLIM interactions with mRFP‐RTN3 and mRFP‐RTN6 or without an acceptor (−) as a negative control are shown. (a) The raw FRET‐FLIM data. (b) Pseudo‐coloured lifetime maps display the lifetime values for each point within the region of interest. (c) The distribution of lifetimes across the entire image with blue shades representing longer GFP fluorescence lifetimes than green ones. (d) Representative decay curves of a single point (indicated by blue cross‐hairs in a,b) with an optimal single exponential fit, where χ2 values from 0.9 to 1.2 are considered an excellent fit to the data points [file MPP-23-1807-s004.pdf]
