# Supplementary figures and images for: Reticulons 3 and 6 interact with viral movement proteins
Source: Mol Plant Pathol. 2022 Aug 20;23(12):1807–14. doi: 10.1111/mpp.13261 (PMC9644274; doi:10.1111/mpp.13261)

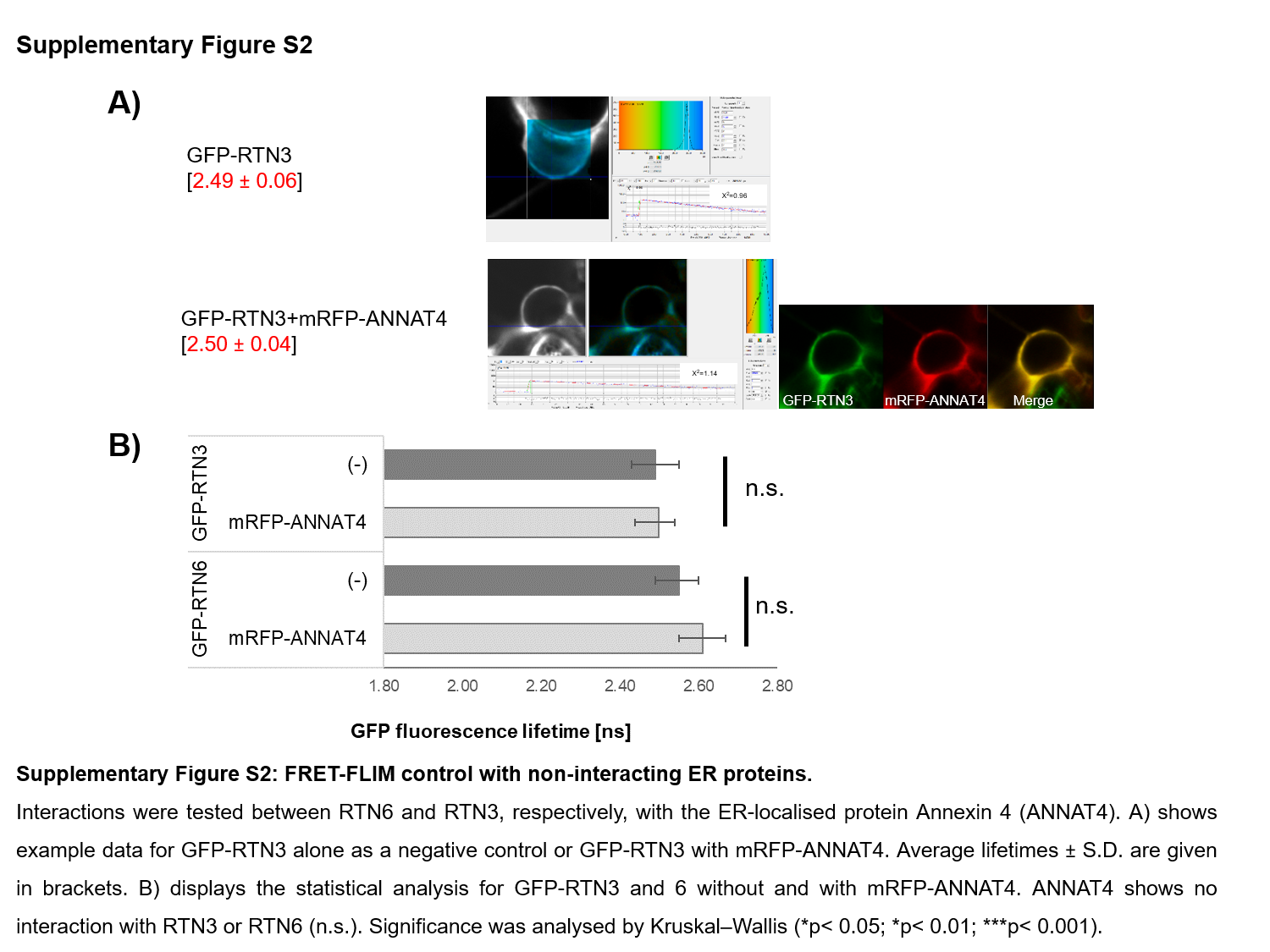

Supplement: Supplementary file 2 — Figure S2 FRET‐FLIM control with noninteracting endoplasmic reticulum (ER) proteins. Interactions were tested between RTN6 and RTN3, respectively, with the ER‐localized protein Annexin 4 (ANNAT4). (a) Example data for GFP‐RTN3 alone as a negative control or GFP‐RTN3 with mRFP‐ANNAT4. Average lifetimes ± SD are given in brackets. (b) Statistical analysis for GFP‐RTN3 and GFP‐RTN6 without and with mRFP‐ANNAT4. ANNAT4 shows no interaction with RTN3 or RTN6 (n.s.). Significance was analysed by the Kruskal–Wallis test (*p < 0.05, *p < 0.01, ***p < 0.001) [file MPP-23-1807-s003.tif]

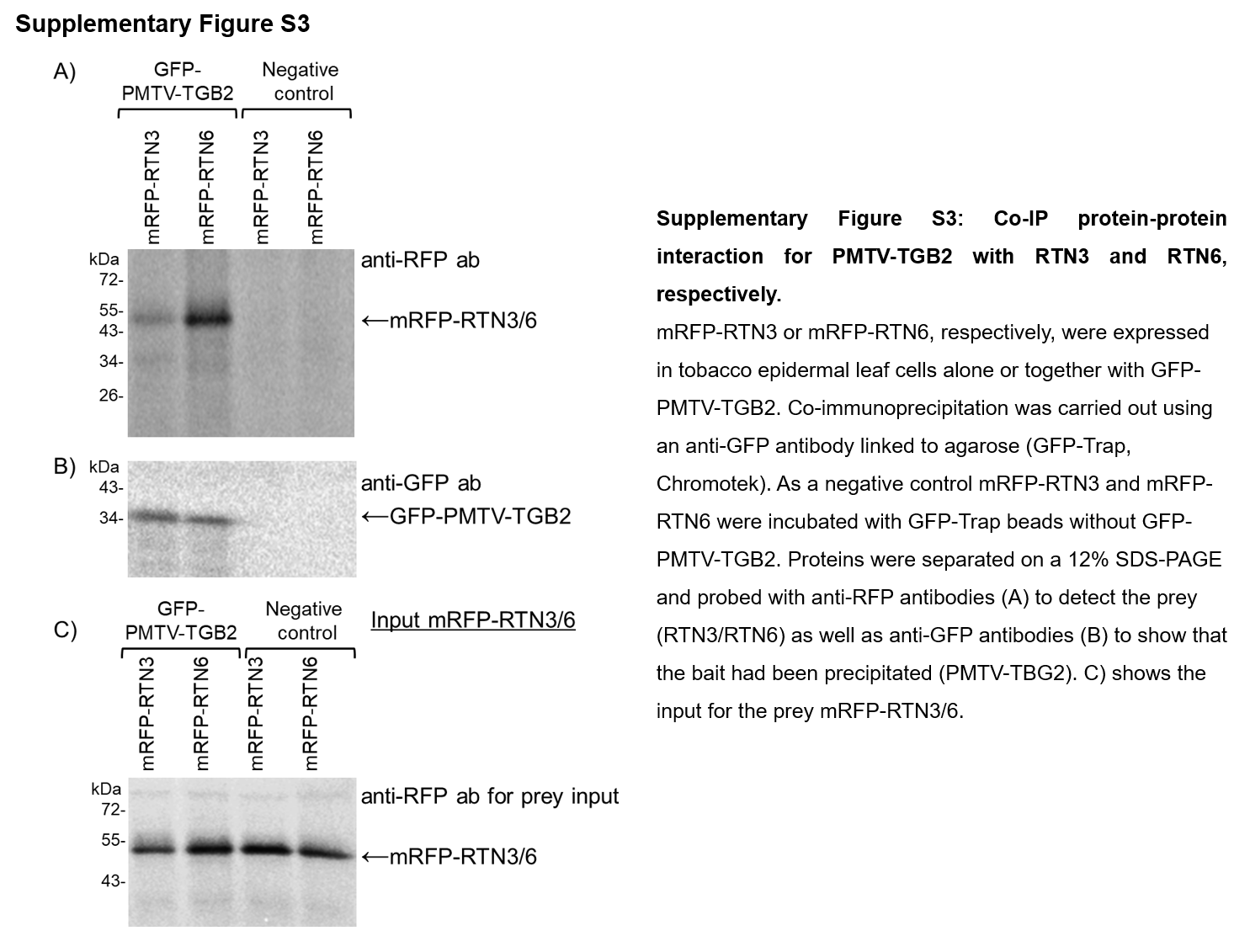

Supplement: Supplementary file 3 — Figure S3 Coimmunoprecipitation assay to determine protein–protein interactions for PMTV‐TGB2 with RTN3 and RTN6. mRFP‐RTN3 or mRFP‐RTN6 was expressed in tobacco epidermal leaf cells alone or together with GFP‐PMTV‐TGB2. Coimmunoprecipitation was carried out using an anti‐GFP antibody linked to agarose (GFP‐Trap; Chromotek). As a negative control mRFP‐RTN3 and mRFP‐RTN6 were incubated with GFP‐Trap beads without GFP‐PMTV‐TGB2. Proteins were separated by 12% SDS‐PAGE and probed with anti‐RFP antibodies (a) to detect the prey (RTN3/RTN6) as well as anti‐GFP antibodies (b) to show that the bait had been precipitated (PMTV‐TBG2). (c) The input for the prey mRFP‐RTN3/6 [file MPP-23-1807-s005.tif]

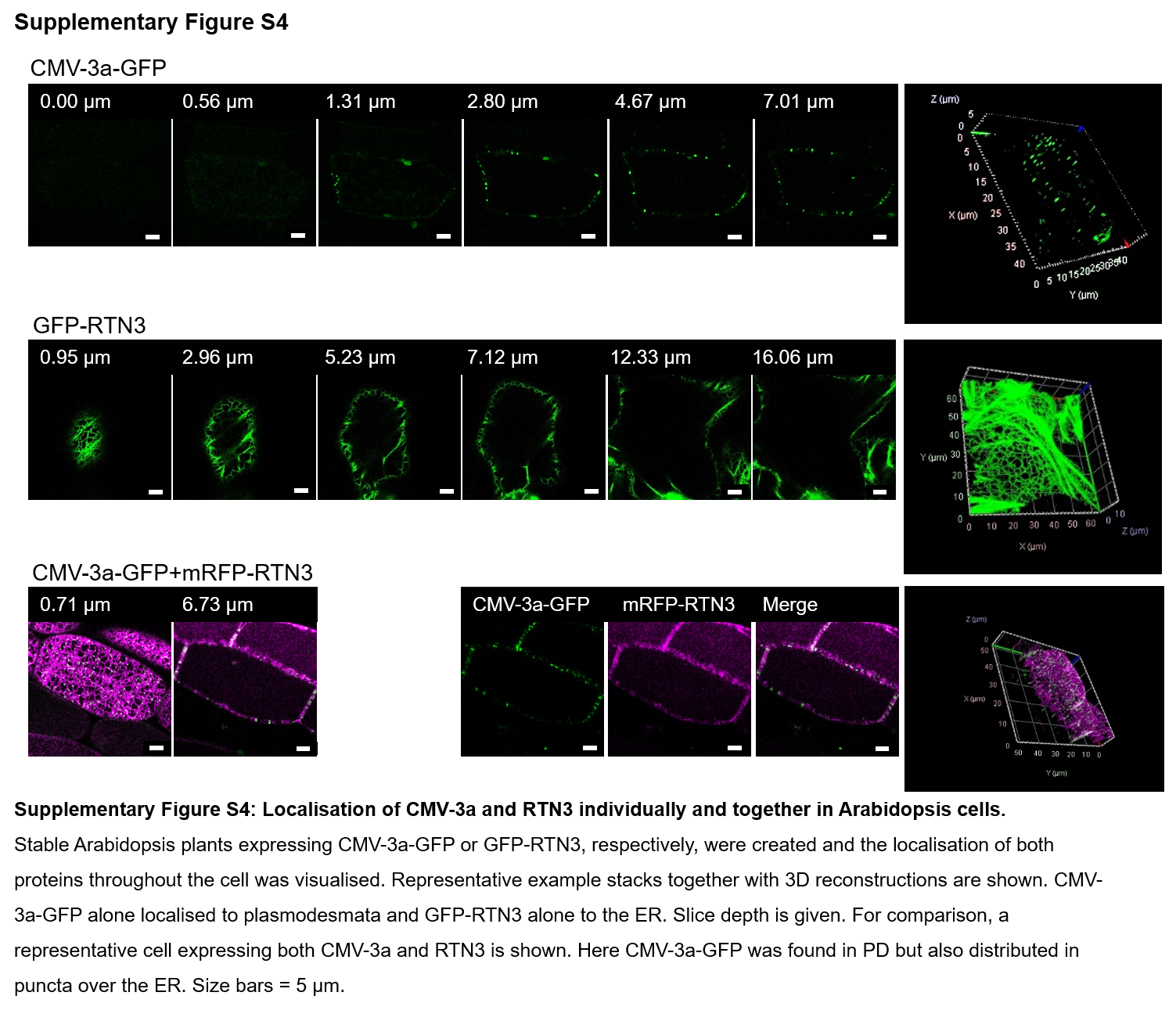

Supplement: Supplementary file 4 — Figure S4 Localization of CMV‐3a and RTN3 individually and together in Arabidopsis cells. Stable Arabidopsis plants expressing CMV‐3a‐GFP or GFP‐RTN3 were created and the localization of both proteins throughout the cell was visualized. Representative example stacks together with 3D reconstructions are shown. CMV‐3a‐GFP alone localized to plasmodesmata (PD) and GFP‐RTN3 alone to the endoplasmic reticulum (ER). Slice depth is given. For comparison, a representative cell expressing both CMV‐3a and RTN3 is shown. Here CMV‐3a‐GFP was found in PD but also distributed in puncta over the ER. Size bars = 5 μm [file MPP-23-1807-s001.tif]
