# Supplementary material for: Reticulons 3 and 6 interact with viral movement proteins
Source: Mol Plant Pathol. 2022 Aug 20;23(12):1807–14. doi: 10.1111/mpp.13261 (PMC9644274; doi:10.1111/mpp.13261)
Supplement: Supplementary file 5 — Figure S5 Transient coexpression of CMV‐3a‐GFP and mRFP‐RTN3 in tobacco epidermal leaf cells. CMV‐3a‐GFP (green) was coexpressed in tobacco epidermal leaf cells with mRFP‐RTN3 (magenta) and the localization was analysed in n = 4 biological replicas with 10 cells each. Representative example images are shown in (a–f). In this system, CMV‐3a‐GFP localized to plasmodesmata (PD) (a,b) only but not the endoplasmic reticulum (ER) (c) in 68% of cells visualized. In 32% of cells, CMV‐3a‐GFP was additionally also localized in dots on the ER (d,e), labelling both the peripheral ER and some PD (f). Size bars = 5 μm for (a–d) and = 10 μm for (f) [file MPP-23-1807-s002.pdf]

Supplementary Figure S5a

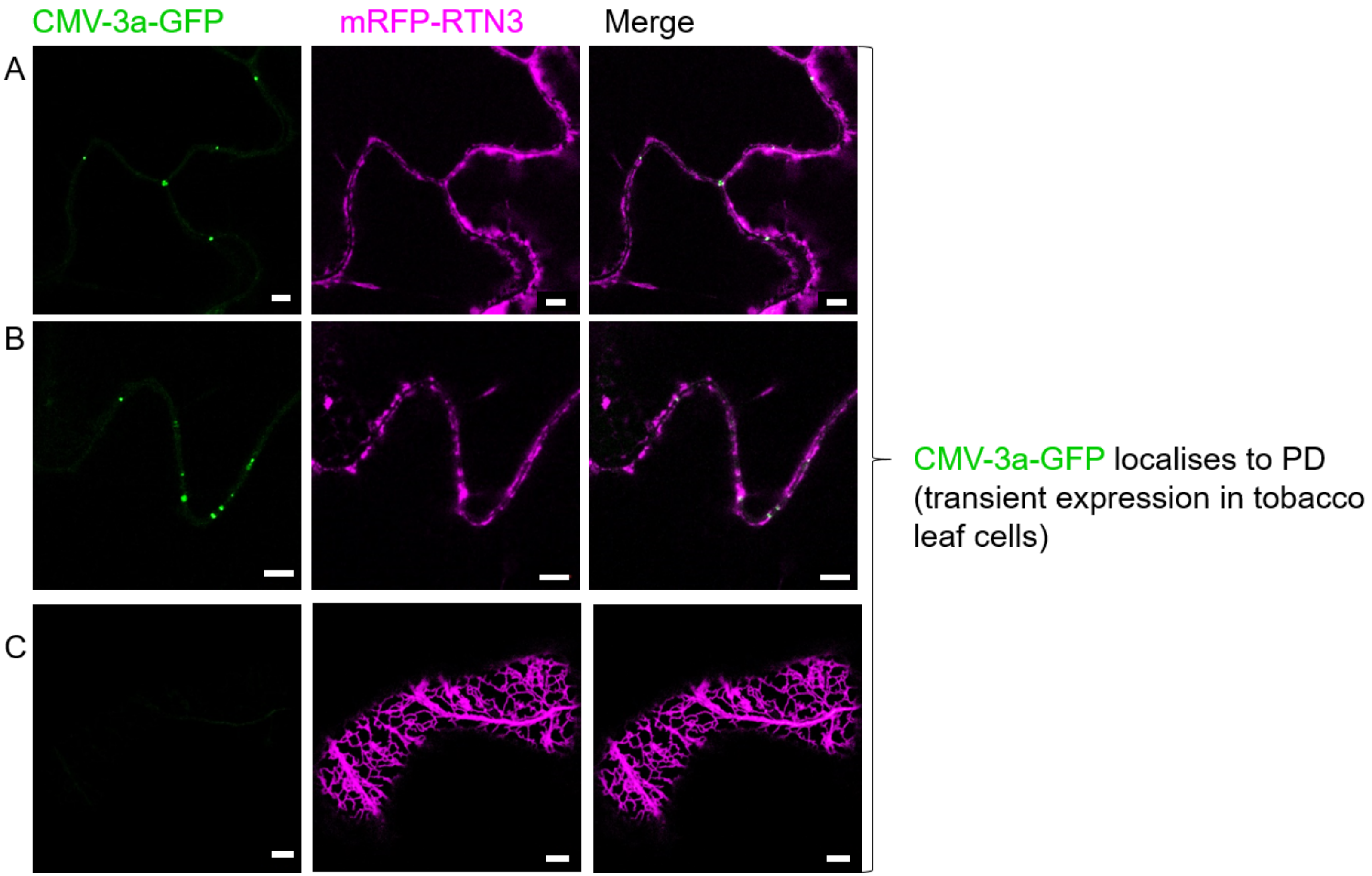

Supplementary Figure S5: Coexpression of CMV-3a-GFP and mRFP-RTN3 transiently in tobacco epidermal leaf cells. CMV-3a-GFP (green) was coexpressed in tobacco epidermal leaf cells with mRFP-RTN3 (magenta) and the localisation analysed in n=4 biological replicas with 10 cells each. Representative example images are shown (A-F). In this system, CMV-3a-GFP localised to PD (A, B) only but not the ER (C) in 68% of cells visualised. In 32% of cells, CMV-3a-GFP was additionally also localised in dots on the ER (D, E), labelling both the peripheral ER and some PD (F). Size bars = 5 µm for A-D and = 10 µm for F.

Supplementary Figure S5b

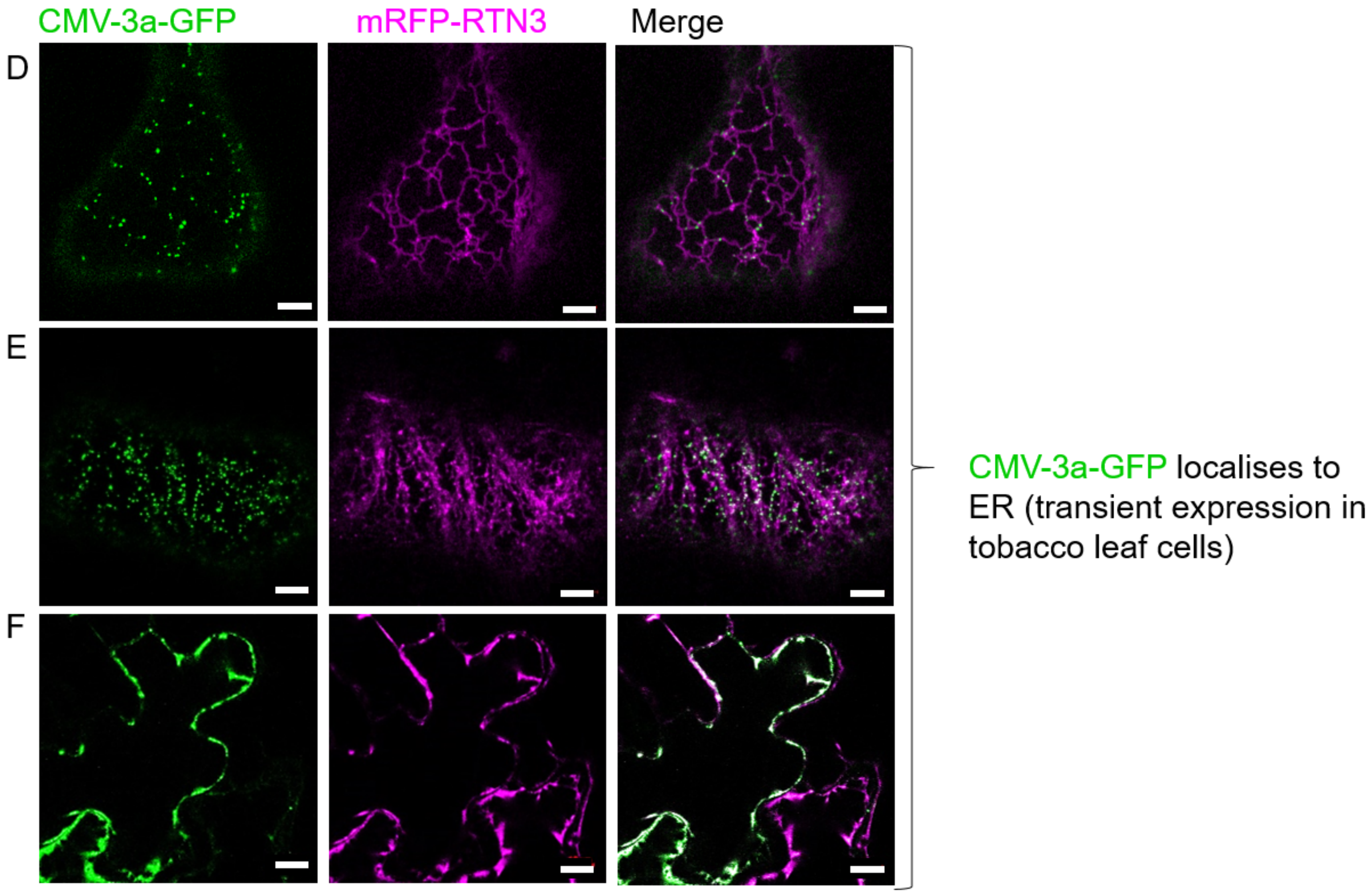

Supplementary Figure S5: Coexpression of CMV-3a-GFP and mRFP-RTN3 transiently in tobacco epidermal leaf cells. CMV-3a-GFP (green) was coexpressed in tobacco epidermal leaf cells with mRFP-RTN3 (magenta) and the localisation analysed in n=4 biological replicas with 10 cells each. Representative example images are shown (A-F). In this system, CMV-3a-GFP localised to PD (A, B) only but not the ER (C) in 68% of cells visualised. In 32% of cells, CMV-3a-GFP was additionally also localised in dots on the ER (D, E), labelling both the peripheral ER and some PD (F). Size bars = 5 µm for A-D and = 10 µm for F.
