# Supplementary material for: Reticulons 3 and 6 interact with viral movement proteins
Source: Mol Plant Pathol. 2022 Aug 20;23(12):1807–14. doi: 10.1111/mpp.13261 (PMC9644274; doi:10.1111/mpp.13261)
Supplement: Supplementary file 6 — Text S1 Materials and methods used in this work [file MPP-23-1807-s006.docx]

**Text S1: Materials and methods used in this work**

Constructs

RTN3- and RTN6-mRFP fusion constructs (Knox *et al.*, 2015; Kriechbaumer *et al.*, 2015) and GFP fusions of PVX TGB2, and PVX TGB3 have been previously described (Tilsner *et al.*, 2012). PMTV TGB2, BSMV TGB2, CMV-3a and TMV-30k were amplified with Gateway adapters, recombined into pDONR201 entry vector and their sequence verified. Correct clones were then recombined with pGWB405 or 406 (Nakagawa *et al.*, 2007) to produce C- or N-terminal GFP fusions, respectively. CMV-3a-GFP and CMV-3a-RFP were produced in the vectors pB7FWG2 or pB7WGR2, respectively (Karimi, De Meyer and Hilson, 2005).

Mating-based Split-Ubiquitin System (mbSUS)

The mating-based Split-Ubiquitin System (mbSUS) was carried out according to (Grefen, Obrdlik and Harter, 2009). In brief: genes of interest were cloned into the vector pMetYC-Dest (bait; RTN3 and 6) or pNX32-Dest (prey; vMP), respectively. The yeast strains THY.AP4 (bait strains) and THY.AP5 (prey strains) were inoculated in 5 ml YPD medium (2% peptone, 2% glucose, 1% yeast extract, 2% agar; pH = 6.0) and incubated shaking overnight at 30°C. 2 ml of the overnight cultures were transferred to fresh 100 ml YPD and incubated shaking for 3–5 h until the cultures reached an OD_600_ of 0.5–0.8. Cells were harvested by centrifugation for 10 min at 2,000×g and the supernatant was discarded. The pellet was then washed with 20 ml sterile dH_2_O, the centrifugation step repeated and the supernatant discarded. Cells were resuspended in 1 ml of 0.1 M lithium acetate (LiAc) and transferred to a 2 ml Eppendorf tube. The cells were pelleted by centrifugation for 2 min at 1,000×g and the supernatant discarded. The pellet was again resuspended in 200 μl 0.1 M LiAc (sufficient for 10 transformations) and incubated at room temperature for 30 min. For each transformation 10 μl of ssDNA (10 mg/ml pre-boiled) and 5 μl of plasmid DNA (approx. 200 ng/μl) were mixed with 70 μl of 50 % PEG, 10.5 μl of 1 M LiAc, 1.5 µl of ssDNA and 18 μl of competent yeast.

The mix was incubated at 30°C for 30 min using a PCR machine. After a heat shock at 43°C for 15 min, cells were spun down at 2,000×g for 5 min and the supernatant discarded. The pellet was washed with 100 μl sterile dH_2_O, spun down and the supernatant discarded. Cells were resuspended in 100 μl sterile dH_2_O, plated on the appropriate selection plates (SC-ADE^+^, HIS^+^, TRP^+^, URA^+^ for THY.AP4 and SC-ADE^+^, HIS^+^, LEU^+^ for THY. AP5) and incubated for 3 days at 30°C.

SC-minimal media: 1.7 g/l yeast nitrogen base (without amino acids), 5 g/l ammonium sulphate, 20 g/l glucose, 1.5 g/l of SC-dropout mix, 20g/l agar; pH to 6.0.

Auxotrophy selection chemicals: ADE: 0.2 g of adenine sulphate (10 ml per litre media); URA: 0.2 g of uracil (10 ml per litre media); LEU: 1.0 g of L-leucine (10 ml per litre media); TRP: 1.0 g of L-tryptophan (2 ml per litre media).

For mating, pMetYC-Dest/THY.AP4 bait colonies (RTN3 and 6, respectively) were grown overnight at 30° in 3 ml of SC-ADE^+^, HIS^+^, TRP^+^, URA^+^ media. pNX32-Dest/THY.AP5 prey fusions were grown overnight at 30°C in 5 ml SC-ADE^+^, HIS^+^, LEU^+^. Cells were pelleted and resuspended in YPD (20 µl of YPD for each mating). 20 µl of every required bait and prey combination were mixed in a 96-well-plate and 4 µl of each mating were immediately dropped onto a YPD plate. The plate was incubated overnight at30°C. A replicator stamp with a sheet of sterile velvet was used to transfer the colonies onto SC-ADE^+^, HIS^+^ plates. After 3 days at 30°C, the cells were again transferred using the replicator stamp onto SC-minimal media and grown for 4 days. Growing cells show a positive reporter gene activity indicating an interaction of the corresponding bait and prey fusion proteins. pNubWt-Xgate containing the prey Nub wildtype protein was included as a positive control.

Tobacco plant material and transient protein expression in tobacco leaf cells

For transient protein expression, 5-week-old tobacco (‘Petit Havana’) plants grown in the greenhouse were used. *Agrobacterium tumefaciens*-mediated transient expression was carried out according to (Sparkes *et al.*, 2006). In brief, each expression vector was introduced into the *A. tumefaciens* strain GV3101 by heat shock transformation. Transformants were grown overnight in 5 mL of YEB medium (5 g/l beef extract, 1 g/l yeast extract, 5 g/l sucrose, and 0.5 g/l MgSO_4_∙7H_2_O) supplemented with the antibiotics for the vector and rifampicin to select for agrobacteria. After overnight shaking at 25°C, 1 mL of the bacterial culture was pelleted by centrifugation at 2,500g for 5 min at room temperature. The pellet was washed twice with 1 ml of infiltration medium (50 mM MES, 2 mM Na_3_PO_4_∙12H_2_O, 0.1 mM acetosyringone, and 5 mg m/l glucose) and then resuspended in 1 mL of infiltration buffer. The suspension was diluted to a final optical density at 600 nm of 0.1 and pressed through the stomata on the lower epidermal surface using a 1-ml syringe. Transformed plants then were incubated under normal growth conditions for 72 h.

FRET-FLIM Data Acquisition

Discs from infiltrated tobacco leaves were excised, and FRET-FLIM data acquisition and analysis was performed according to (Schoberer and Botchway, 2014; Kriechbaumer *et al.*, 2015) using a two-photon microscope at the Central Laser Facility of the Rutherford Appleton Laboratory. In brief, data acquisition was carried out using a two-photon microscope built around a Nikon TE2000-U inverted microscope combined with a modified Nikon EC2 confocal scanning system to allow for multiphoton FLIM (Botchway *et al.*, 2015). Laser light at a wavelength of 920 nm was produced by a mode-locked titanium sapphire laser (Mira; Coherent Lasers), producing 200-fs pulses at 76 MHz, pumped by a solid-state continuous wave 532-nm laser (Verdi V18; Coherent Laser). To illuminate specimens on the microscope stage, the laser beam was focused to a diffraction limited spot through a water-immersion objective (Nikon VC; 360, numerical aperture of 1.2). Fluorescence emission was collected without descanning and passed through a BG39 (Comar) filter in order to block the near-infrared laser light. Line, frame, and pixel clock signals were generated and synchronized with an external detector in the form of a fast microchannel plate photomultiplier tube (Hamamatsu R3809U). Raw FLIM data was generated by linking these via a time-correlated single-photon-counting PC module SPC830 (Becker and Hickl). Prior to FLIM data collection, the GFP and mRFP expression levels in the plant samples within the region of interest were confirmed using a Nikon EC2 confocal microscope with excitation at 488 nm and 543 nm, respectively. A 633-nm interference filter was used to minimize the contaminating effect of chlorophyll autofluorescence emission that would otherwise obscure the mRFP and GFP emissions. Data were analysed by obtaining excited-state lifetime values of a region of interest, and calculations were made using SPCImage analysis software version 5.1 (Becker and Hickl). The distribution of lifetime values within the region of interest was generated and displayed as a curve. Only values that had a χ^2^-value between 0.9 and 1.4 were taken for analysis. The median lifetime value and minimum and maximum values for one-quarter of the median lifetime values from the curve were used to generate the range of lifetimes per sample (Supplementary Figure S1). For each combination, at least three biological samples with a minimum of ten technical replicates were used for the analysis. Average lifetimes and standard deviations were calculated for each combination. A reduction in excited-state lifetime of 0.2 ns is indicative of energy transfer and is therefore considered a positive protein-protein interaction (Stubbs *et al.*, 2005). Significance was analysed by Kruskal–Wallis (*p< 0.05; *p< 0.01; ***p< 0.001).

Co-IP protein-protein interaction and Western Blot analysis

GFP-PMTV-TGB2 was co-expressed in tobacco epidermal leaf cells with mRFP-RTN3 or mRFP-RTN6, respectively. Co-immunoprecipitation was carried out using an anti-GFP antibody linked to agarose (GFP-Trap, Chromotek) according to the manufacturer’s’ protocol. Proteins were separated on a 12% SDS-PAGE, transferred to blotting membrane and probed with anti-RFP antibodies (Proteintech) to detect the prey (RTN3/RTN6) as well as anti-GFP antibodies (Proteintech) together with anti-mouse (anti-RFP) and anti-rabbit (anti-GFP) secondary antibodies (Proteintech) to show that the bait had been precipitated. Primary antibodies (dilution 1:1000) were incubated overnight, secondary antibodies (dilution 1:1000) for 1 hour.

Transformation and imaging in *Arabidopsis thaliana* seedlings

Floral dipping of Arabidopsis was performed as recommended by (Clough and Bent, 1998). In short, pelleted transformed agrobacteria were re-suspended in a 5% sucrose, 500 µl/l Silwet 77 solution. *Arabidopsis thaliana* Col-0 flowering stems were dipped into this culture and then agitated for approximately 1 min. The plants were wrapped in cling film for 24 hours to ensure survival of the agrobacteria. This process was repeated 7 days later to increase the efficiency of transformation. After an additional week, watering was ceased and the plants were dried in preparation for seed collection. Once dried and collected, transformed seeds were identified by selection MS plates containing BASTA at 5 µg/ml. Transformants were transferred to soil at approximately 10 days old and grown under greenhouse conditions.

For imaging Arabidopsis seeds were sterilized using 70% ethanol for 3 minutes, then grown on sterile 1/2 MS plates (2.4g/l Murashige and Skoog medium, 0.8% phytoagar in dH_2_O, pH 5.6-5.8). Seeds were kept in the dark for 3 days at 4°C to ensure uniform germination, and then transferred to a growth cabinet with standard conditions. Images were taken using a Zeiss 880 laser scanning confocal microscope with Airyscan detector using a Zeiss PlanApo 100x/1.46 NA oil immersion objective. An excitation wavelength of 488nm was used to observe GFP and 561nm for RFP.

**References:**

Botchway, S. W., Scherer, K. M., Hook, S., Stubbs, C. D., Weston, E., Bisby, R. H. and Parker, A. W. (2015) 'A series of flexible design adaptations to the Nikon E-C1 and E-C2 confocal microscope systems for UV, multiphoton and FLIM imaging', *J Microsc,* 258(1), pp. 68-78.

Clough, S. J. and Bent, A. F. (1998) 'Floral dip: a simplified method for Agrobacterium-mediated transformation of Arabidopsis thaliana', *Plant J,* 16(6), pp. 735-43.

Grefen, C., Obrdlik, P. and Harter, K. (2009) 'The determination of protein-protein interactions by the mating-based split-ubiquitin system (mbSUS)', *Methods Mol Biol,* 479, pp. 217-33.

Karimi, M., De Meyer, B. and Hilson, P. (2005) 'Modular cloning in plant cells', *Trends Plant Sci,* 10(3), pp. 103-5.

Knox, K., Wang, P., Kriechbaumer, V., Tilsner, J., Frigerio, L., Sparkes, I., Hawes, C. and Oparka, K. (2015) 'Putting the Squeeze on Plasmodesmata: A Role for Reticulons in Primary Plasmodesmata Formation', *Plant Physiol,* 168(4), pp. 1563-72.

Kriechbaumer, V., Botchway, S. W., Slade, S. E., Knox, K., Frigerio, L., Oparka, K. and Hawes, C. (2015) 'Reticulomics: Protein-Protein Interaction Studies with Two Plasmodesmata-Localized Reticulon Family Proteins Identify Binding Partners Enriched at Plasmodesmata, Endoplasmic Reticulum, and the Plasma Membrane', *Plant Physiol,* 169(3), pp. 1933-45.

Nakagawa, T., Suzuki, T., Murata, S., Nakamura, S., Hino, T., Maeo, K., Tabata, R., Kawai, T., Tanaka, K., Niwa, Y., Watanabe, Y., Nakamura, K., Kimura, T. and Ishiguro, S. (2007) 'Improved Gateway binary vectors: high-performance vectors for creation of fusion constructs in transgenic analysis of plants', *Biosci Biotechnol Biochem,* 71(8), pp. 2095-100.

Schoberer, J. and Botchway, S. W. (2014) 'Investigating protein-protein interactions in the plant endomembrane system using multiphoton-induced FRET-FLIM', *Methods Mol Biol,* 1209, pp. 81-95.

Sparkes, I. A., Runions, J., Kearns, A. and Hawes, C. (2006) 'Rapid, transient expression of fluorescent fusion proteins in tobacco plants and generation of stably transformed plants', *Nat Protoc,* 1(4), pp. 2019-25.

Stubbs, C. D., Botchway, S. W., Slater, S. J. and Parker, A. W. (2005) 'The use of time-resolved fluorescence imaging in the study of protein kinase C localisation in cells', *BMC Cell Biol,* 6(1), pp. 22.

Tilsner, J., Linnik, O., Wright, K. M., Bell, K., Roberts, A. G., Lacomme, C., Santa Cruz, S. and Oparka, K. J. (2012) 'The TGB1 Movement Protein of Potato virus X Reorganizes Actin and Endomembranes into the X-Body, a Viral Replication Factory', *Plant Physiology,* 158(3), pp. 1359-1370.
